# Contributions des tradipraticiens de santé au traitement antirétroviral : Étude de cas à Nampula, Mozambique

**DOI:** 10.4102/phcfm.v10i1.1031

**Published:** 2018-10-31

**Authors:** Paulo H. das Neves Martins Pires, Abdoulaye Marega, José M. Creagh

**Affiliations:** 1 Health Sciences Faculty, Lúrio University, Mozambique

## Abstract

**Contexte:**

L’épidémie du virus de l’immunodéficience humaine au Mozambique est un problème grave de santé publique et le Ministère de la Santé a étendu le traitement antirétroviral à tous les districts du pays. Cependant, on constate un nombre élevé d’abandon du traitement encore insuffisamment évalué. L’Organisation Mondiale de la Santé recommande que les tradipraticiens de santé collaborent avec les systèmes nationaux de santé dans les pays en développement, pour combattre cette épidémie, mais il existe peu d’actions dans ce domaine à ce jour.

**Objectif:**

Évaluer la connaissance des tradipraticiens sur l’infection et leur disponibilité à coopérer avec les services de santé dans la Province de Nampula au Mozambique, pour améliorer les résultats du traitement antirétroviral.

**Lieux:**

Cinq centres de santé des districts de la Province de Nampula, au Nord du Mozambique, avec des taux élevés d’incidence du virus d’immunodéficience humaine et d’abandon du traitement.

**Méthodes:**

Une étude mixte transversale, utilisant des interviews ciblés et des discussions de groupes focaux. Les données quantitatives étaient traitées par fréquence et les données qualitatives par analyse de discours et ethnographie locale.

**Résultats:**

Nous avons interviewé 79 tradipraticiens de santé. La perte de poids était souvent considérée comme le signal principal de suspicion d’infection par le virus d’immunodéficience humaine et certains tradipraticiens ne pas les signes de la maladie ; la majorité pensait que les antirétroviraux améliorent la qualité de vie des patients, ne prétendait pas traiter l’infection, savait qu’elle n’est pas curable, avait une idée sur le concept de bonne adhésion au traitement et référait les cas compliqués au centre de santé. En ce qui concerne l’alimentation, la moitié considérait exclusivement les céréales comme l’aliment principal ; les fruits étaient importants pour un quart ; l’eau potable est ignorée. La majorité était prête à collaborer avec le système de santé et avait des propositions de coopération pratique : la qualification et la reconnaissance individuelle et la formation intégrée avec les professionnels de santé.

**Conclusion:**

Les tradipraticiens connaissaient l’infection par le virus d’immunodéficience humaine et les facteurs associés, mais il y a des lacunes. Ils ont signalé qu’ils utilisaient principalement les plantes médicinales, ce qui peut contribuer au traitement des infections opportunistes et la majorité référait déjà des patients au centre de santé ; mais la collaboration nécessite une procédure éducative et une articulation structurée. Les lacunes de connaissance empêchent une coopération efficace dans le combat contre l’épidémie. Le groupe est disponible pour coopérer avec le système de santé pour améliorer les résultats du traitement antirétroviral, mais pour ça il est nécessaire d’informer et former les tradipraticiens dans un processus intégré de collaboration avec les professionnels de santé conventionnels.

## Introduction

Le Sud de l’Afrique est la région du monde la plus affectée par l’infection au virus de l’immunodéficience humaine (VIH), avec environ 25.6 millions des personnes infectées en 2016 et 2/3 des nouvelles infections.^1^

La Déclaration de Politique sur le VIH et le Syndrome de l’Immunodéficience Humaine Acquise (SIDA) de l’Assemblée des Nations Unies de 2011^2^, ont formulé des recommandations aux Gouvernements pour s’engager à atteindre l’accès universel à la prévention et au traitement et le renforcement des capacités des communautés pour soutenir la rétention des malades et améliorer l’adhésion au traitement, pour arrêter l’épidémie du VIH.

L’adhésion au traitement antirétroviral (TARV) constitue le facteur clé du degré et de la durée de la suppression virale.^3^ Une étude réalisée en Zambie en 2011 a montré que les réseaux sociaux de proximité avaient un impact significatif sur l’adhérence des jeunes malades au TARV.^4^

Une autre étude en Afrique du Sud, a montré que les Tradipraticiens de Santé (TPS) constituaient une ressource de santé utile mais sous-utilisée, qui pouvait soutenir le système biomédical et réduire l’impact de l’infection au VIH. Les TPS offrent des soins de santé culturellement adaptés à la prévention de l’infection au VIH et peuvent référer les cas positifs aux centres de Santé (CS).^5^ C’est dans les communautés rurales que le TPS intervient le plus utilement en conseillant soit les sujets séropositifs, soit les personnes qui ont développé la maladie, donnant des conseils et des directives utiles pour l’utilisation des remèdes disponibles.^6^

En 2010 le Mozambique avait 1.6 millions de porteurs du VIH et environ 500 000 patients en TARV (35%), comparativement aux 3500 patients en traitement en 2003, ce qui prouve l’effort du Ministère de la Santé qui a rendu disponible le TARV dans tous les districts du pays. Durant les trois dernières années, l’abandon du traitement augmente, présentant un nouveau défi de santé publique, à cause du danger de résistance aux antirétroviraux (ARV).

Dans la province de Nampula, le taux de prévalence du VIH se trouve en dessous des 10% (moyenne nationale 16%).^7^ Pourtant, l’abandon du TARV peut augmenter la transmission de l’infection et provoquer la dissémination de virus résistants augmentant la mortalité. La population mozambicaine a déjà souffert d’un impact très négatif provoqué par cette infection et un des défis est la surcharge du système de santé, qui souffre déjà d’une carence généralisée de professionnels. Le niveau de connaissance de la population sur le VIH est élevé mais le changement de comportement est encore insignifiant.

Dès le début du programme de lutte contre le SIDA, suivant les recommandations de l’Organisation Mondiale de la Santé (OMS), les services nationaux de santé essayent d’établir une relation de coopération avec les TPS, surtout dans les pays à faibles ressources. Cependant, jusqu’à présent, les actions qui considèrent et incluent réellement les TPS sont presque inexistantes.^8^ Actuellement l’accès au Système National de Santé (SNS) et à la médecine conventionnelle moderne se limite à environ 40% de la population tandis que presque la totalité des Mozambicains font appel à la médecine traditionnelle. C’est pour cette raison qu’ils fréquentent les TPS pour résoudre les problèmes de santé.^9^ Dans les endroits où les deux systèmes sont disponibles, les personnes ont recours à la médecine traditionnelle et à la médecine moderne.^10^ Les TPS peuvent être des éducateurs efficaces sur le VIH dans les communautés, avec leur capacité de diffuser des messages préventifs, en utilisant des témoignages personnels, des histoires et des proverbes.^11^

Pour accomplir une couverture universelle et efficiente du TARV, il faut comprendre les systèmes qui fonctionnent le mieux, pour intervenir sur les déterminants de santé locaux d’une population déterminée.^12^ Au Mozambique, des pratiques socioculturelles traditionnelles (scarification rituelle, comportement sexuel après décès du partenaire), favorisent la dissémination de l’infection par le VIH. Ces aspects doivent être identifiés et transformés en pratiques préventives. Pourtant, le manque d’études sur les alternatives de pratiques traditionnelles préventives et l’absence de stratégies socioculturelles dans les interventions de santé, peuvent contribuer au taux élevé de prévalence du VIH.

Ce travail de recherche vise à établir une ligne de base pour permettre la transformation d’aspects culturels négatifs en pratiques positives, pour prévenir la transmission du virus et améliorer le programme du TARV, selon les recommandations du Plan d’Accélération de la Prévention, Diagnostique et Traitement du VIH/SIDA.^13^ Face aux défis rencontrés, notre problématique est la suivante : La connaissance des TPS sur l’infection au VIH, sur son traitement et leur collaboration avec le système national de santé, pourraient-elles contribuer à accroître l’efficacité du TARV?

Ainsi l’objectif de cette étude est d’évaluer la connaissance des TPS sur l’infection a VIH et leur disponibilité à coopérer avec les services de santé dans la Province de Nampula au Mozambique, pour améliorer le programme de lutte contre le VIH et/ou SIDA.

Les objectifs spécifiques sont les suivants : (1) décrire les connaissances des TPS sur l’infection au VIH, (2) évaluer leur disponibilité et leurs propositions pour coopérer avec le SNS.

## Méthodes

Étude descriptive transversale mixte (avec utilisation de la méthodologie qualitative et quantitative).

La consultation des documents statistiques a permis l’identification des districts cibles dans la Province de Nampula. Nous avons d’abord fait l’analyse des données de l’abandon du TARV à travers les registres de la Direction Provinciale de la Santé de Nampula et nous avons aussi interviewé les responsables du programme TARV.

Des interviews structurées ont permis l’évaluation des connaissances des TPS sur l’infection et son traitement. Les discussions en groupes focaux ciblent les propositions de coopération avec le SNS pour améliorer les résultats du programme VIH et/ou du SIDA.

Le dialogue avec les acteurs locaux a respecté les principes de la communication interculturelle.

Population de l’étude : TPS et accoucheuses traditionnelles dans la zone de couverture des CS avec service TARV, dans les districts de la province de Nampula pendant la période d’étude.

Groupe d’étude : vingt participants ont été invités, en respectant la parité de genre, par le responsable de l’association professionnelle du district et par le responsable de liaison avec les TPS de chacun des CS des cinq capitales des districts (Lalaua, Mossuril, Murrupula, Nacarõa, Nampula).

Critères d’inclusion : CS des districts avec incidence du VIH parmi les femmes enceintes (10%) et taux d’abandon du TARV (20%, malgré qu’il soit largement sous-estimé dans les statistiques des CS), supérieurs à la moyenne provinciale, classifiés dans les catégories géographiques du programme VIH/SIDA : ville, campagne, corridor, littoral ; volontariat des participants et signature du terme de consentement éclairé.

Critères d’exclusion : TPS en état d’intoxication alcoolique ou qui manifestaient le désir de ne pas participer à l’étude ou de l’abandonner.

Variables géographiques : district, association professionnelle.

L’analyse du discours et l’ethnographie, constituant l’orientation méthodologique de l’étude, ont été réalisées manuellement dans les registres des discussions des groupes, séparément par les trois chercheurs et discutées par la suite.^14^ Les thèmes principaux et les codes de données ont été choisis par les chercheurs pour répondre aux questions de l’étude: (1) pratique traditionnelle (procédure de formation, temps de pratique, maladies traitées, traitement utilisé), (2) connaissance sur le VIH (formation en VIH, concept d’infection au VIH, traitement du VIH, importance du TARV, concept d’adhésion thérapeutique), (3) connaissance des facteurs associés (concept de tuberculose (TB), traitement de la TB, nourriture), (4) coopération avec le SNS (formation, référence, accès). Les interviews ont été préalablement testées avec un groupe de dix TPS appartenant à un autre district.

Les données collectées lors des interviews et des discussions ont été introduites dans le programme informatique SPSS^21^ et ont été analysées par fréquence des codes, pour chaque thème et centre de santé et présentés dans des tables.^15^

La collecte de données a été réalisée de septembre à novembre 2014. Les chercheurs ont collecté les données dans les installations des CS des capitales des cinq districts. Ils ont conduit les interviews face à face pendant environ 20 minutes et les groupes de discussion focaux pendant 90 min, avec l’aide d’un traducteur choisi par les TPS. Les chercheurs étaient médecins avec une pratique de terrain, professeurs à la Faculté des Sciences de la Santé (FSS), avaient de l’expérience dans cette méthodologie, n’étaient pas connus des participants et n’avaient pas de préjugées négatifs ou positifs sur les TPS. La collecte de données a été précédée d’une présentation au groupe de TPS des objectifs de l’étude en langue locale, dans une salle du CS ou une autre salle disponible ailleurs, et de l’explication du terme de consentement éclairé. Les interviews ont été menées en privé dans la langue préférée du participant (Portugais ou Macua) et les réponses écrites sur un formulaire en papier ont été relues aux participants pour confirmer les données recueillies. On n’a pas répété les interviews. On a terminé avec une discussion de groupe focal (cinq groupes avec de 11 à 20 participants), avec traduction simultanée Portugais – Macua, avec un animateur qui posait les questions et assurait la participation de tous les TPS, respectant la parité de genre et un responsable des registres en papier (on n’a pas fait d’enregistrement audio ou vidéo des discussions), pour identifier la disponibilité et les conditions de coopération avec le SNS; tous les 79 TPS interviewés ont participé dans les discussions focales. A la fin de la discussion les informations écrites ont été présentées aux participants pour confirmer leur accord.^16^

Nous avons considéré comme facteur de risque la fiabilité des réponses aux interviews et on a appliqué une triangulation des questions pour éviter des biais de collecte de données. Les résultats ont été confirmés par les participants après lecture des réponses en langue maternelle pour éviter des biais de conclusion. Les données recueillies ont été revues par les trois chercheurs séparément avant leur introduction dans la banque informatique pour éviter des biais d’analyse.

La FSS de l’Université Lúrio a obtenu un financement du Fonds de Développement Institutionnel du Ministère de l’Education pour la réalisation de cette étude et un partenariat avec l’Association des Médecins Traditionnels du Mozambique (AMETRAMO) pour la sélection des TPS.

## Considération éthique

Le protocole de l’étude a été approuvé par le Comité Institutionnel de Bioéthique pour la Santé de l’Université Lúrio et respecte la Déclaration d’Helsinki (révision 2013) 02/CBISUL.03/14, Mars 27, 2014.

## Résultats

Tous les TPS invités présents dans le CS le jour de la collecte de données, ont participé aux interviews et aux discussions focales. Nous avons interviewé 79 TPS dans cinq districts, 62% de sexe masculin avec un âge moyen de 38 ans : 14 à Lalaua, 20 à Mossuril, 15 à Murrupula, 11 à Nacarõa et 19 à Nampula.

L’analyse quantitative a montré que tous les TPS étaient inscrits dans une association professionnelle (95% chez AMETRAMO) et que la majorité (92%) pratiquaient aussi dans d’autres districts. En ce qui concerne la procédure de formation, la majorité (59%) déclarent : (1) à la suite d’un rêve (100% à Lalaua) ; (2) par formation pratique auprès d’un autre TPS, 24% ; (3) 15% référent un épisode de maladie comme moment de révélation de la vocation et de la capacitation ; (4) 10% étaient détectés par un autre TPS à la naissance.

Selon le temps d’expérience professionnelle, la majorité des TPS (65%) ont plus de 10 ans ; le groupe avec 5 à 10 ans de pratique est prévalent à Mossuril et Nacarõa; le groupe le moins expérimenté se localise à Mossuril et Murrupula.

Les TPS traitent un vaste éventail de maladies; une grande partie de celles-ci ne cadrent pas avec les modalités diagnostiques de la médecine conventionnelle et les quelques désignations ‘conventionnelles’ utilisées ne correspondent pas à la pathologie référée; 51% rapportent traiter des problèmes de ‘sorcellerie’. Quant au type de pratiques thérapeutiques, 76% des TPS utilisent des plantes médicinales et 55% utilisent aussi les ‘esprits’ des progéniteurs.

En ce qui concerne la connaissance sur l’infection au VIH, la perte de poids, associée ou non à d’autres signes, est considérée comme le principal indice de l’infection (33%,) suivie des ‘boutons’ (24%) ; 25% des TPS ne connaissent pas les signes de la maladie, notamment à Nacarõa (50%) et à Lalaua (20%) ; 56% avaient une formation spécifique sur le VIH :

‘… quand je vois un voisin qui maigrit vite, je pense qu’il a attrapé l’infection.’ (TPS Masculin, 42 ans, Nampula)

La grande majorité (97%) répondent que les ARV améliorent la qualité de vie des patients, 96% rapportent ne pas traiter définitivement le VIH, 88% savent que cette infection n’a pas de cure actuellement :

‘*…* les malades qu’initient le traitement récupèrent assez vite.’ (TPS masculin, 51 ans, Mossuril)

‘*…* je peux aider une personne infectée, mais je sais que je ne peux pas guérir la maladie, *…*.’ (TPS Féminin, 38 ans, Nacarõa)

La majorité des TPS (85%) ont une idée approximative des caractéristiques d’une bonne adhésion au TARV mais 15% (tous résidents à Nacaroa) ne connaissent pas ce concept :

‘*…* être adhérant, ça veut dire prendre les comprimés tous les jours et à la même heure …’ (TPS Masculin, 35 ans, Lalaua)

En ce qui concerne la connaissance sur la TB, 61% désignent la toux comme symptôme pathognomonique mais 19% ne connaissent pas la maladie, surtout à Nacarõa. Une partie importante dit traiter la TB (51%), surtout à Nampula (33%), à Lalaua (25%) et à Murrupula (23%) :

‘… je sais traiter un patient avec une toux persistante avec mes plantes médicinales…,’ (TPS Masculin, 28 ans, Nampula)

Par rapport à la coopération avec la médecine conventionnelle, beaucoup d’entre eux (85%) transfèrent les cas compliqués aux CS :

‘*…* si j’ai un patient avec une maladie difficile, je l’envoie à l’hôpital *…*’ (TPS Féminin 54 ans, Mossuril)

Par rapport à l’alimentation, 48% considèrent exclusivement les céréales comme l’aliment principal ; les fruits sont considérés comme un aliment important pour 23% ; à Lalaua et à Nacarõa, les protéines et les graisses ne sont même pas mentionnées. L’eau potable est ignorée dans tous les districts.

En ce qui concerne l’analyse qualitative quant à la coopération avec le SNS, la grande majorité suggèrent l’identification et caractérisation individuelle des TPS dans les CS :

‘J’aimerais que les professionnels du CS sachent ce que je traite et combien de personne j’aide tous les jours.’ (TPS Masculin, 35 ans, Murrupula)

‘…, il serait bon d’avoir une carte d’identification pour être reconnu quand tu vas au CS.’ (TPS Féminin, 34 ans, Lalaua)

Ils recommandent la formation conjointe avec les professionnels de santé :

‘*…,* si on peut avoir une formation sur les maladies chroniques avec les professionnels du CS, on va être reconnus et il y aura un respect mutuel.’ (TPS Féminin, 49 ans, Mossuril)

Ils demandent la reconnaissance quand ils ont accès aux services de santé et des gants et masques pour quelques malades ; ils soulignent aussi l’importance de diffuser l’information sur le thème VIH/TB :

‘*…* nous aidons les malades qui vont à l’hôpital en leur expliquant ce qu’il faut faire, mais quand on a besoin d’y être traité, on doit attendre trop longtemps pour la consultation *…’* (TPS Féminin, 40 ans, Lalaua)

‘J’envoie les cas difficiles à l’hôpital, mais je n’ai jamais reçu d’information des infirmières *…*’ (TPS Féminin, 36 ans, Nampula)

‘Vous devriez demander aux prêtres de nos églises de bien informer sur ces maladies.’ (TPS Masculin, 36 ans, Nampula)

Les propositions de coopération des TPS avec le SNS sont résumées dans le Case 1.

**CASE 1:** Propositions des tradipraticiens de santé pour la coopération avec le Service National de Santé.

Identifier et caractériser les capacités des TPS dans chaque Centre de Santé.
Formation avec les professionnels de santé, permettant la reconnaissance mutuelle et la création d’une relation de partenariat et coopération.
Accès facilité aux services de santé (carte d’identification).
Fournir l’équipement de protection individuelle (gants, masques) pour quelques maladies.
Quand ils détectent chez un patient le VIH ou la TB, ils le transfèrent aux CS et lui conseillent de ne pas abandonner le traitement.
Ils doivent aussi recevoir des patients envoyés par les professionnels de la santé.
Les professionnels de la santé ne doivent pas blâmer les patients quand ils recourent d’abord aux TPS.
Les TPS qui orientent les patients dans le CS et contribuent à la communication de messages d’éducation sanitaire, doivent être reconnus par les professionnels de la santé.
Ils conseillent la divulgation du contenu des formations, qu’ils considèrent comme très importantes, pour transmettre les informations dans des écoles, les églises, les mosquées, les associations et les communautés.

TPS, Tradipraticiens de santé ; VIH, Virus de imunodeficience humaine ; TB, Tuberculose ; CS, Centre de santé

## Discussion

Les TPS ont des connaissances sur l’infection au VIH, le TARV, la TB et les facteurs associés, mais ils ont des lacunes importantes (l’alimentation par exemple). Ils coopèrent déjà avec le système de santé conventionnel, mais ne se sentent pas reconnus ou motivés par celui-ci. Pourtant ils ont des propositions pour développer la coopération.

Ce groupe de TPS utilise majoritairement les plantes médicinales pour leurs traitements, un résultat similaire à celui trouvé dans une étude réalisée au Ghana^17^ ce qui peut contribuer au traitement des infections opportunistes et augmenter l’immunité de ces patients.^18^ Ils réfèrent les cas compliqués au SNS, ce qui est confirmé par une étude faite en Afrique du Sud^19^, ils se montrent absolument disponibles pour coopérer avec le SNS et présentent plusieurs propositions.

Une étude réalisée dans la province de Zambèze au Mozambique^20^, a montré que les TPS ont un âge moyen plus élevé que la population en général et un niveau de scolarité inférieur. Ils pratiquent fréquemment la scarification rituelle, c’est-à-dire qu’ils utilisent des lames pour couper la peau des patients et introduire des mélanges de plantes dans la blessure qui saigne. Cette étude, contrairement à la nôtre, montre que la majorité des TPS ne référent pas les patients au CS, principalement pour deux raisons : (1) ils ne connaissent pas les signes et les symptômes du patient infectés par le VIH, (2) ils croient pouvoir traiter eux-mêmes cette maladie. Majoritairement, ils croient que la cause de la maladie est spirituelle et non infectieuse. Des formations précédentes sur le VIH ne semblent pas être associées à une meilleure connaissance de la maladie ou à des références plus effectives. Dans notre étude, la majorité des TPS affirment être intéressés par une collaboration avec les professionnels du SNS, de même que les TPS dans une étude au Mali.^21^ La formation des TPS avec une procédure intégrée a déjà été recommandée par d’autres auteurs^22^ : (1) les TPS collaborent avec les services de santé modernes, (2) des structures locales sont créées pour faciliter l’accès aux services de santé, (3) les TPS participent à des études et des recherches avec les professionnels de santé^23^, (4) le succès de cette démarche dépend d’une bonne identification des TPS avec connaissance et disponibilité, ainsi que de l’établissement d’une relation solide avec les professionnels, en manifestant de l’intérêt, du respect professionnel, et de la confiance; il faudra aussi assurer la garantie d’un prix juste en échange de leur temps et de leurs plantes, ainsi que les droits de propriété intellectuelle personnels et collectifs.^24,25,26^

Les TPS constituent un groupe de participants locaux avec un impact potentiel considérable, dans la prévention et le traitement des maladies chroniques au Mozambique. Ils montrent aussi un désir de développement éducatif et sont prêts à la coopération, pour la couverture universelle des soins de santé. Reste à trouver les interlocuteurs.

Le fait que les TPS participants résident majoritairement autour des villes capitales des districts, a limité notre étude (biais d’échantillon) ; aussi, les interviews n’ont pas réussi à différentier ceux qui disent ‘traiter’ le VIH ou la TB de ceux qui pensent les ‘soigner’, ce qui représente une ambivalence dans les réponses. L’interprétation ethnographique et conventionnelle des pathologies et des traitements des TPS n’a pas été traitée ici et reste un vaste domaine de recherche à poursuivre.

## Conclusion

Même si les TPS ont une certaine connaissance de l’infection au VIH et des facteurs associés, nous avons constaté beaucoup de lacunes. Les TPS sont entièrement disponibles à collaborer avec le SNS, par exemple, pour réduire le taux d’abandon du TARV, pour établir un système de référence avec le SNS et en conséquence diminuer la mortalité et la morbidité des porteurs de VIH et l’incidence de l’infection au VIH dans la population. Pour cela, il est nécessaire de qualifier et de former les TPS dans une procédure intégrée avec les professionnels de la santé et de reconnaître et de renforcer leur rôle de collaborateurs avec les services de santé modernes.
